# “Looking at Myself in the Future”: how mentoring shapes scientific identity for STEM students from underrepresented groups

**DOI:** 10.1186/s40594-020-00242-3

**Published:** 2020-08-18

**Authors:** Kaitlyn Atkins, Bryan M. Dougan, Michelle S. Dromgold-Sermen, Hannah Potter, Viji Sathy, A. T. Panter

**Affiliations:** 1 Department of Psychology and Neuroscience, University of North Carolina at Chapel Hill, Chapel Hill, NC USA; 2 Social and Behavioral Interventions Program, Department of International Health, Johns Hopkins Bloomberg School of Public Health, Baltimore, MD USA; 3 Department of Anthropology, University of North Carolina at Chapel Hill, Chapel Hill, NC USA; 4 Department of Sociology, University of North Carolina at Chapel Hill, Chapel Hill, NC USA

**Keywords:** Mentoring, Science identity, Diversity, Post-secondary

## Abstract

**Background:**

Mentorship has been well-established in the literature as fostering scientific identity and career pathways for underrepresented minority students in science, technology, engineering, and mathematics (STEM) fields. Mentorship is prioritized by programs that aim to increase diversity and support future leadership in STEM fields, but in-depth understanding of mentorship in these contexts remains limited. Drawing on qualitative interview data, we sought to understand the relationship between mentoring and scientific identity among a diverse sample of 24 students in one such program, in order to inform program development.

**Results:**

Qualitative analysis of the data revealed that mentorship, especially research mentorship, was common and played a role in formation of scientific identity. Students with research mentors tended to say they strongly identified as scientists, whereas those who lacked research mentorship varied in their level of scientific identity. In interviews, research-mentored students described mentors as colleagues who gave them opportunities to grow and as examples to look up to. Students valued mentors with whom they identified on the basis of demographic similarity or shared values, as well as those who challenged them in their academic and research endeavors.

**Conclusions:**

Our analysis highlights how different mentoring experiences can contribute to development of future STEM leadership. We discuss implications for practice, including the need for tailored mentoring approaches and research-focused mentoring, and offer several recommendations for research and programming.

## Introduction

The USA has a long-standing goal of improving and maintaining diversity in the science, technology, engineering, and mathematics (STEM) workforce (Olson & Riordan, 2012; Institute of Medicine, 2011). While improvements in the participation of women and minorities in STEM have been made, STEM retention and degree attainment are persistently lower for underrepresented minority (URM; Black/African American, Hispanic/Latinx, and American Indian, Alaska Native, or Native Hawaiian) and female students compared with male, white, and Asian students (Estrada et al., 2018). These disparities persist at undergraduate and graduate levels (Estrada et al., 2018; Ciocca Eller & DiPrete, 2018). To reduce this gap, scholars have highlighted the importance of early preparation (including K-12 STEM preparation), increased access to and awareness of STEM education, college affordability, commitment of leadership to STEM diversity and inclusion, and ongoing academic and social support for diverse STEM students (Institute of Medicine, 2011).

As academic institutions strive to create diverse and inclusive environments in STEM, a number of strategies have been adopted to facilitate student retention, timely degree completion, and career goal-setting for URM and female students. One strategy to improve URM and female participation in STEM is the use of STEM enrichment programs, formal university-led programs that are designed to foster scientific community, expose students to research and mentorship, and improve academic and career outcomes (Domingo et al., 2019; Tsui, 2007). These programs often combine financial assistance with academic and social support. In this analysis, we explore the experiences of students in one such program, with a focus on mentoring and scientific identity. Specifically, we seek to answer the following questions: (1) How do high-performing URM and female STEM students characterize successful mentoring relationships? (2) How do mentoring relationships influence students’ scientific identities?

### Theoretical Framework

This study draws on identity theory as an underlying framework for analysis and interpretation of findings. According to identity theory, when individuals claim an identity, they attribute to themselves a set of meanings about their role, group membership, or unique personhood (Burke & Stets, 2009). These meanings are then communicated to others through behaviors and social interactions; individuals interpret the responses of others to those interactions as approval or disapproval, and change or control their identities. This identity process includes first seeing oneself as having a particular identity, then experiencing verification from others of one’s identity, and ultimately viewing an identity as prominent or salient (Burke & Stets, 2009; Stets et al., 2017). Identities with greater salience, or a higher position on a hierarchy of all one’s identities, are thought to be more persistent over time (Serpe, 1987; Serpe & Stryker, 2011).

In the STEM literature, the science identity has been widely studied (Simpson & Bouhafa, 2020). The importance of scientific identity salience in shaping outcomes has been well-demonstrated in this literature. Quantitative results from a national study of undergraduate science majors indicated that the salience of students’ scientific identities directly impacted graduate school matriculation and mediated the impact of STEM enrichment programs on graduate school matriculation (Merolla & Serpe, 2013). Scholars have further suggested that for female and URM students, given their underrepresentation in STEM fields, the development of scientific identity and its salience over time may be especially important and should be prioritized to facilitate increased participation and retention of these students in STEM fields (Lee, 1998; Merolla & Serpe, 2013).

Verification of identity is also important (Simpson & Bouhafa, 2020; Stets et al., 2017). The verification process occurs in small groups of peers, through interactions with mentors, and through self-reflection (Riley & Burke, 1995). Discrepancies between how STEM students see themselves and how they think others see them can shape their later behaviors; individuals will behave in ways to reassert their own identities to others, respond to perceived identity threat, and reduce discrepancies. Stets et al. (2017) suggest that if others see students as “better” scientists than students themselves, students may be less likely to continue in science. They further suggest this may be because the high expectations of others, in contrast to students’ expectations of themselves, are distressing; students may then act in ways to lower the expectations of others. In contrast, if students perceive that others do not see them as scientists, they may behave in targeted ways to reassert their identities as scientists, in order to raise others’ expectations and respond to identity threat.

Because mentors play an important role in this identity verification process, which in turn influences identity salience, it is critical to better understand the complexities of their relationships with mentees and the ways in which mentor-mentee interactions take shape.

### Scientific Identity

Previous research has shown that a student’s development of scientific identity (i.e., considering oneself a scientist) improves academic performance, retention and persistence in STEM, and STEM degree completion (Maton et al., 2016; Merolla & Serpe, 2013; Syed et al., 2011). One qualitative study, following women of color from their undergraduate training into their science careers, found that women’s science identity trajectories were meaningfully shaped by receiving recognition as a scientist from others, including mentors (Carlone & Johnson, 2007). Similarly, a national study of STEM persistence with students who indicated they planned to major in a STEM field during their freshman year found that URM students with higher academic self-concept were more likely to persist in STEM fields (Chang et al., 2014).

One strategy for fostering scientific identity and increasing its salience among STEM undergraduates is mentoring. Particularly among URM students in STEM, mentoring has been shown to foster scientific identity and STEM career pathways (Summers & Hrabowski, 2006). A 2007 review of strategies to increase diversity in STEM found that mentoring programs are widely utilized and can lead to higher grade point averages (GPAs), increased self-efficacy, and more clearly defined academic goals (Tsui, 2007). A recent review of factors contributing to academic success among Latinx STEM majors similarly identified mentoring, including peer mentoring, as a key driver of success for this population (Winterer et al., 2020).

Research also suggests that different types of mentoring may differentially influence identity development. For example, Aikens et al. (2017), in their research with undergraduate life sciences researchers, found that mentoring structures explained observed differences in scientific identity among women, men, and URM students. Similarly, a study of mentoring structures among mentored undergraduate life sciences researchers found that students with a direct relationship to a faculty mentor, compared to those whose relationships to faculty were mediated by postgraduate researchers (graduate students or postdoctoral scholars), had increased scientific identity (Aikens et al., 2016).

Studies have also found that mentor-mentee dyads with shared beliefs, values, and backgrounds are preferred by mentors and mentees, and may yield more positive outcomes (Aikens et al., 2017; Eby et al., 2004; Eby et al., 2008; Eby et al., 2000). An assessment of students who were engaged in undergraduate research at a public minority-serving institution in the USA found that socioemotional and culturally relevant mentoring were strongly correlated with development of research skills and independence, both key elements of scientific identity (Haeger & Fresquez, 2016). In this study, socioemotional mentors were perceived as warm and friendly, good listeners, and positive role models. Culturally relevant mentors were perceived to understand their mentee’s ethnic, gender, and class background and to closely relate to that background.

The pathways through which mentoring fosters scientific identity have been an area of recent exploration in the higher education literature. However, multiple longitudinal quantitative evaluations of mentored STEM students have shown that mentoring influences scientific identity as mentors link students to career resources and research opportunities, provide emotional support, foster students’ confidence and science self-efficacy, and facilitate their valuing of scientific research (Estrada et al., 2018; Byars-Winston et al., 2015). A quantitative study with minority undergraduate researchers found that, across intersectional race/ethnicity × gender subgroups, research self-efficacy was significantly associated with scientific identity and was shaped by research and mentoring experiences (Byars-Winston & Rogers, 2019). Similarly, a mixed-methods study with undergraduate mentor-mentee dyads found that students who received instrumental and socioemotional support from their mentors had higher scientific identity, and that negative mentoring experiences were associated with lower scientific identity (Robnett et al., 2018). These findings suggest that mentoring may provide students with diverse forms of support, research skills and independence, and science self-efficacy which shape their scientific identities (National Academies of Sciences, Engineering, and Medicine, 2019).

However, despite this recent evidence, there is a continued need for in-depth, qualitative understanding of the processes linking mentoring to scientific identity, including how these processes may differ for students of different backgrounds.

### Successful Mentoring Relationships in STEM

In their 2009 review, Crisp and Cruz (2009) suggest that mentoring relationships can be broadly characterized as (1) focusing on growth and development of an individual (often referred to as the protégé); (2) providing a broad range of support, including professional and psychological support; and (3) being characterized as being “personal and reciprocal” in nature, although some mentoring relationships are becoming less personal with new technologies that allow for Internet-based mentorship. For the purposes of this study, we conceptualized mentoring as a formal or informal relationship between a more experienced person (mentor) and a less-experienced person (protégé), characterized by provision of support, with a focus on professional and personal growth and development (Hernandez et al., 2017). While mentoring relationships can vary in length, we conceptualize them as relationships that are sustained beyond a one-time encounter, involving months or even years of repeated contact between mentor and protégé.

In current research on mentoring among undergraduate STEM students, the role of mentor has been distributed among a broad range of individuals, including faculty members, staff members, graduate students or postdoctoral scholars, peers, friends, community members, and family. Mentoring relationships can be formal (planned and organized through structured programs or interventions) or informal (emerging through existing relationships or outside of structured programs) in nature, though some literature suggests informal relationships may be more successful (Crisp & Cruz, 2009; Hernandez et al., 2017; Tsui, 2007). However, whether mentoring relationships are formal or informal, it is clear that they should involve intentionality on the part of both mentor and mentee in identifying goals of the relationship (National Academies of Sciences, Engineering, and Medicine, 2019).Prolonged mentoring relationships have also been shown to yield better outcomes, compared with those only lasting a summer or single academic term (Haeger & Fresquez, 2016).

While demographic similarity to mentors may play a role in forming quality relationships, some scholars have suggested that the success of mentoring is more likely to hinge on the extent to which protégés view themselves as similar to mentors in terms of their values and beliefs than on the extent to which mentors and protégés “match” each other culturally or ethnically. A study of mentoring experiences among high-performing URM undergraduate seniors majoring in STEM disciplines found that the most important factor associated with a mentee’s determination of the relationship as high-quality was perceived (vs. actual) similarity to a mentor; that is, the extent to which mentees felt they shared values (Hernandez et al., 2017). Importantly, this phenomenon occurred regardless of actual demographic similarities between mentors and mentees. Others have highlighted that, because URM mentors are often more limited in number (and because mentoring URM students should not be the responsibility of URM faculty or scholars alone), demographically matching mentor-mentee dyads may be infeasible, and that matching dyads based on shared values and beliefs may be more important (National Academies of Sciences, Engineering, and Medicine, 2019).

Researchers have further argued that the qualities of mentoring *relationships*, and not the characteristics of mentors themselves, are what matter for mentees. Key theoretical attributes of successful mentoring relationships include developing research skills and self-efficacy, building trusting relationships, developing science identity and belonging, advancing equity and inclusion, fostering independence, and actively advocating for mentees (Pfund et al., 2016).

### Present Study

While the positive impacts of mentoring for STEM students have been well-established in the quantitative literature, few papers have explored in-depth how this occurs. Our analysis aims to fill this gap by using qualitative data to explore the complexities of mentoring relationships and to identify potential pathways through which mentoring shapes scientific identity, among a diverse sample of high-performing STEM undergraduates.

In this study, we examine mentorship relationships in the Chancellor’s Science Scholars (CSS), a scholarship program designed to increase the number of URM individuals who obtain doctorates in science. CSS at The University of North Carolina at Chapel Hill (UNC-CH) is modeled after the Meyerhoff program of the University of Maryland at Baltimore County (Summers & Hrabowski, 2006) and aims to increase diversity and support future leadership in STEM (Domingo et al., 2019). CSS aims to facilitate scientific identity by creating a community of student scientists and engaging students in research.

A core CSS program value is mentorship, which is facilitated both formally and informally through dedicated CSS program advisors, faculty and postgraduate research mentors, and university academic professional advisors. Students also find mentoring outside CSS through peers, friends, and community members. While CSS does not formally match students with mentors on the basis of demographic or other characteristics, understanding mentor-mentee dynamics for CSS students will inform future CSS program development and help program administrators identify mentoring strategies that can best support students and foster positive STEM outcomes. Further, given CSS was modeled after a program at a different institution with a different student population, continuous evaluation of program elements, including mentoring, is critical to ensure continued program success and may inform STEM enrichment programs at other institutions (George et al., 2019).

## Method

### Institution and Program Background

In order to achieve its goal of increasing diverse future leadership in STEM, UNC-CH started the CSS program in 2013 with support from Howard Hughes Medical Institute and the UNC-CH Office of the Chancellor. UNC-CH is a highly ranked four-year public research university, with over 90% of the incoming 2013 class (3,946 students) reporting GPAs of 4.0 or higher (UNC-CH Office of Undergraduate Admissions, 2013). It is a predominately white university; approximately 63% of students in the incoming 2013 class identified as White or Caucasian only, per federal reporting guidelines. In the same class, 59% of incoming students were female, 18% were first-generation students, and 36% were recipients of need-based financial aid (UNC-CH Office of Undergraduate Admissions, 2013).

CSS recruits high-achieving students from across the USA who apply to the program during their senior year of high school. Students are selected based on their academic merit and commitment to STEM careers, social justice, and diversity and inclusion (Domingo et al., 2019). Since 2013, CSS has enrolled an annual cohort of approximately 40 students who live together for their first year at UNC-CH and receive an annual merit scholarship, research lab placements, and professional support including graduate school application assistance, extra tutoring, and test preparation. To remain in CSS, students must participate in a rigorous summer program, maintain a 3.0 GPA in an approved STEM major, stay actively involved in the program, take two STEM courses per semester, join a research lab, write a research thesis, and apply to four doctoral programs.

### Data Collection

Each year that CSS students are enrolled in the program, they participate in end-of-year interviews focused on students’ overall experiences at UNC-CH and in CSS. A standard, semi-structured interview guide is used for all interviews, which covers a range of topics about coursework, classroom experiences, research, mentorship, scientific identity, and post-graduation goals and plans. Interviews are used rather than focus group discussions, as they allow program evaluators to understand the personal experiences and perspectives of individuals in-depth.

While mentorship was not the sole focus of the interviews used for this analysis, we asked each student if they had a relationship with a mentor, how that relationship was characterized, and how it influenced their other experiences. Similarly, we asked each student about their identity as a scientist, and probed for additional thoughts and experiences related to this identity as they came up throughout the interview. All interviews were conducted by trained graduate research assistants and were audio-recorded and professionally transcribed. Because this analysis aimed to understand students’ mentorship experiences and scientific identities during the time that they prepared to transition out of college, only interviews conducted with CSS students in their senior years were included in our analytic sample (*n* = 24).

Ethics approval for this study was obtained from the Office of Human Research Ethics at the University of North Carolina-Chapel Hill. Prior to conducting interviews, written informed consent was obtained from each participant.

### Data Analysis

We thematically analyzed the data, using Dedoose (2019), a Web-based qualitative data analysis software, to organize and code transcripts. First, we read each interview once for content; during this read of the data, we noted key topics, descriptive details, and patterns in the interview to get a sense of the student’s experience as a whole. In a second read of each interview, we noted patterns and themes across interviews; each member of the team independently noted these and organized them into thematic groups, which were then collectively restructured and consolidated into a codebook. We used a combination of deductive coding, wherein a priori codes were developed based on the interview guide, and inductive coding, wherein additional codes were added to reflect themes discovered in the data. Coding was conducted iteratively.

First, we independently coded a total of six randomly selected interviews, meeting throughout this process to discuss coding strategies and revise code definitions before coding the remaining data. To assess intercoder reliability, we used an intraclass correlation coefficient (ICC) at the interview level to assess the consistency with which codes were applied at an interview level. We also qualitatively assessed intercoder reliability by independently coding a subset of transcripts and meeting to discuss questions, concerns, and discrepancies in coding strategies. This assessment was performed iteratively until consensus about codes was reached. Representative codes, along with the high-level themes to which they correspond, are summarized in Table 1.

**Table 1:** Summary of relevant codes and corresponding themes

Code	Definition	High-level theme
**Research/lab experience**	Students discuss working in a lab or conducting research (job responsibilities, independent work, experiences presenting or publishing their research).	(1a) Unique role of research mentorship; (2) influence of mentoring on scientific identity
**Relationships with faculty/staff**	Students discuss relationships and interactions with UNC faculty and staff (mentoring, what mentoring means, role models, support received from faculty/staff).	(1) Characterizing successful mentoring relationships
**Part-time jobs/internships**	Students discuss employment and internships: paid/unpaid, summer/academic year, reasons for working (e.g., career development, money), time spent at work/internship, influence on academic and/or CSS experiences.	(2) Influence of mentoring on scientific identity
**Relationships with program staff**	Students discuss interactions with CSS staff, who it is, what their interactions include, such as in advising roles.	(1) Characterizing successful mentoring relationships
**Professional development**	Student discusses opportunities that CSS provides them for furthering their career (providing resources to travel, mentorship, paying for test fees).	(1) Characterizing successful mentoring relationships; (2) influence of mentoring on scientific identity
**Post-graduation motivations and support**	Students discuss their motivations to pursue something post-graduation, instrumental/emotional support they receive toward that goal, and overall preparedness for post-grad life/career.	(1) Characterizing successful mentoring relationships
**Diversity**	Students discuss diversity in STEM, mentorship, CSS, and at UNC more broadly.	(1) Characterizing successful mentoring relationships
**Scientific identity**	Students discuss why they identify/do not identify/are hesitant about identifying as scientists.	(2) Influence of mentoring on scientific identity
**Formative experiences**	Students discuss the personal experiences that shape their identity/absence of their identity as scientists.	(1a) Unique role of research mentorship
**Perceived qualifications**	Students discuss their perceptions and understandings of what it means to be a scientist, including educational requirements, research experience, and professional engagement.	(2) Influence of mentoring on scientific identity

Throughout the analytic process, we used memos to record processes, explore relationships between codes, make comparisons, and expand upon emerging categories and themes (Saldaña, 2009). We also used matrices to summarize data related to codes and emergent categories of interest for this analysis (including mentor roles, mentoring relationship quality, and scientific identity) and to assess trends by demographic or academic characteristics (e.g., major). As themes and categories emerged, the first author continually reviewed transcripts to identify negative cases and pose new questions to the data (Yin, 2011).

### Research Team and Reflexivity

Interviews were conducted by trained graduate research assistants. Interviewers received annual training, wherein they were informed about CSS, the interview protocol, and standards for qualitative interviewing procedures. Interviewers also received ethics training. Interviewers were not involved in analysis of data. Data were coded by three doctoral research assistants (KA, BD, MD, and HP); the first author (KA) led the development of this analysis.

The first author is a White woman who received training in social science and public health at UNC-CH. As an advocate for URM researchers and students in STEM and a scientist and academic with experience as both mentor and mentee, the first author is invested in the potential implications of this study and has assumptions about the nature of mentoring and scientific identity. To clarify these assumptions throughout data analysis, the first author regularly engaged in reflexive memoing and debriefs with the research team. These processes, which included acknowledgement of her own experiences with mentoring and her presuppositions about the challenges faced by URM students in STEM, were used to distinguish the first author’s views and experiences from those of study participants.

## Results

Of the students in our analytic sample, 18 (75%) were female and 13 (54%) were URM (Table 2). Nine respondents (38%) were URM female students. Most students had at least one mentor at UNC-CH. These included research (faculty or postgraduate (graduate students or postdoctoral) researchers, or lab staff), teaching (faculty from whom students took courses), and program mentors (faculty and staff affiliated with CSS). Some students (12.5%) also received mentorship from peers, though this was less common. Importantly, while the CSS program facilitated mentorship for students, many of them found mentorship from other sources. While all students participated in a supervised research experience through CSS, one student mentioned that they did not currently have anyone in their life they considered an active mentor.

**Table 2:** Demographic characteristics of sample (*n* = 24)

Demographic characteristic	***N*** (%)
**Sex**	
Female	18 (75.0)
Male	6 (25.0)
**Underrepresented minority (URM)**	
Yes	13 (54.2)
No	11 (45.8)
**Race**^**a**^	
White/Caucasian	13 (54.0)
Black/African American	6 (25.0)
Asian	2 (8.3)
Native American	2 (8.3)
**Ethnicity**	
Hispanic/Latinx	5 (20.8)
Middle Eastern	1 (4.2)
**Major**^**b**^	
Biological sciences	16 (66.7)
Physical sciences	5 (20.8)
Psychological sciences	3 (12.5)
Mathematics and statistics	3 (12.5)
Computer science	2 (8.3)
**Type of mentorship discussed**	
Research	19 (79.2)
Teaching	7 (29.2)
CSS program	5 (20.8)
Peer	3 (12.5)
Not discussed	1 (4.2)

^a^One student did not report their race

^b^Five students had double majors in STEM fields

We identified two key themes which correspond with our research questions. First, we characterized successful mentorship, highlighting students’ values and preferences in mentoring relationships and the unique role of research mentorship. Second, we identified the influence of mentorship on scientific identity, particularly the role of mentored research in reinforcing mentorship experiences and reinforcing students’ identities.

### Characterizing Successful Mentoring Relationships

Students said they looked to mentors to fill a variety of roles, ranging from intimate involvement with their day-to-day lives on campus to more tangential involvement. Many students had several mentors, each filling a unique role.

I think all of them provide something different … they provide very useful advice, like, different kinds of advice. [With] one of them, [it’s] purely research. He’s like, you know, a connoisseur of research, like what I should do and … how to do that. Another one’s more like life advice … It’s good to have a lot of mentors, just because they can all provide you with different kinds of advice.—Non-URM female

I think I have four or five mentors … I have teachers who have served as mentors for me, just to get through those classes, but also provide a lot of emotional support. And then I also have my mentors, of course, through CSS, who have also provided academic and emotional support as well … my newest mentor, it was just that [researcher], so I’m actually gonna be continuing my work with her in grad school.—URM female

Most students differentiated between advisors and mentors. For these students, advisors were people they sought out when they needed advice, but who remained relatively distant and less involved in their day-to-day college lives. In contrast, mentors—including research, teaching, and program mentors—were not only sources of advice but were intimately involved in their academics, research and other work, and longer-term planning. Importantly, students emphasized the value of having a shared identity with mentors—whether that was a shared racial or ethnic, gender, or other identity. This shared identity was a key feature distinguishing mentors from advisors, and this was particularly salient for URM students. For example, a URM female described how she views her female research mentors differently from a male mentor:

I will consider [male faculty] an advisor more than a mentor. I know they go together, but he’s just – [he will] listen, provide me advice. But the two women, I feel they – I can look up to them to know that I’m going to be where they are one day. So, I just split that up – with [my female mentors] I know I’m going to – I’m looking at myself in the future, and then [my male mentor] is just advice.

Another student, a URM male, found research mentorship through graduate students and postdoctoral researchers. However, his mentoring relationship with a CSS staff member of color served as an important and “inspiring” example to him:

I would list him as a mentor just because he is an African American male that has made it. And he’s doing amazing work and he’s now giving back and continuing to lead more and more minorities in terms of inspiring them to become educational leaders.

Some students intentionally sought out formal mentors who could serve as role models and provide professional and academic support. For example, a URM female student described intentionally seeking out mentorship through a professor because she needed research guidance and support: “I was like, I really want to do research, but I don’t know of anyone that can help me. Then [professor] ended up introducing me to a few people.” While this mentoring was described positively by the student, she and other students emphasized that such relationships were more unidirectional in nature, meaning that students actively sought out support rather than mentors initiating it.

In contrast, many students mentioned that their strongest and most sustained mentoring relationships were more informal or “organic” in nature. One URM male student described an early interaction with a teaching mentor:

So, one day really early in the program we were going to some benefit on campus and it was just a downpour, like think of the hardest rainstorm you’ve ever been in. That was it. We were all dressed up in suits and everything ... I held my umbrella out ...above the doorway and then I just stood in the rain for like five minutes while everybody was walking in and [future mentor] saw that and he thought it was just like cool. And ever since, we’ve been really close … Whenever we run into each other we get coffee. Like so, just organic, you know?

In general, students said they preferred mentoring relationships that felt natural and informal in nature. These relationships also tended to be more bidirectional, meaning both students and mentors took on the role of initiating contact and support. For most students, these informal relationships were characterized by personal closeness, which was not experienced in formal relationships. However, students emphasized that while they valued personal closeness with mentors, they wanted this to be balanced with academic or career accountability. In other words, while students appreciated mentors to whom they related and felt close personally, they felt it was equally important to have mentors who pushed them to achieve. In some mentoring relationships, these qualities were at odds; some students described mentors who challenged them academically but offered little emotional support or intimacy, while others described mentors who were close supporters but did not strongly challenge them. However, students felt that being both emotionally supported and academically challenged was important and made them feel valued as mentees.

Students who lacked the desired level of academic accountability often said their mentorship was missing something. One URM female described how, although she was happy with the personal support received from one of her program advisors, she wished this mentor could support her in other ways:

[It] is more like a personal relationship than academic relationship than I’ve had with a lot of my other CSS advisors ... That being said, sometimes I did wish we talked about – he was not harder on me, but a little bit hard on me of, “What are you doing right now? What do you see yourself doing in a week or two weeks and how are you gonna get those things done?” I don’t really have a mentor or advisor like that. So I kind of have to do it to myself.

Other students received strong accountability from mentors, but felt less comfortable with the mentor if they did not feel close to them personally. Students said it was ideal to find this balance in a single mentor, but if this was not available, they found accountability and closeness through a mix of mentors. For example, a non-URM female described a research mentor that pushed her to achieve her goals, but with whom she did not feel particularly close. For her, as for other students, the desired balance of intimacy and accountability was achieved through a mix of different mentors, including informal peer mentors, rather than from an individual mentor:

[My research mentor is] really great in terms of asking me what I want to do, where I want to go with my life, and how I can reach those goals … But I think sometimes I get more mentorship opportunities out of students or friends that I’m close to, just because I’m more comfortable going to them a little bit more frequently and asking different kinds of questions. So it’s a mix of both.

Similarly, a male respondent described his peer and postgraduate mentors as people who “keep me on top of what I need to be doing,” while faculty and CSS program staff served more personal mentoring roles. The desire for mentoring relationships characterized by shared identity, intimacy, and accountability was expressed by students about all types of mentors. However, as will be elaborated in the next section, research mentors exemplified additional characteristics that differentiated these relationships from others.

#### Unique Role of Research Mentorship

When describing successful mentoring, students often talked about the link between research and mentoring, and cited mentored research experiences as uniquely impactful. Indeed, research mentors were very common in our sample, with 20 of 24 students reporting having at least one research mentor. These were often a students’ direct collaborators on a research project or in their lab, including study principal investigators, graduate students, postdoctoral researchers, or other lab staff. In contrast to teaching or program mentors, who tended to take on more distant, advice-giving roles, students saw research mentors as supportive colleagues who gave them opportunities to grow in their scientific identities and as examples to model.

Important for many students was the link between research mentorship and hands-on research experience. While students found mentors in a variety of places, those more heavily engaged in research activities had not only increased access to mentors, but stronger mentoring relationships overall. Similarly, having a strong mentor seemed to encourage students to become more involved in research activities in their fields. Research mentors’ unique roles as collaborators positioned them well to both advise students and support them in pursuing independent work and exploring new research goals. This type of support played a critical role in giving students confidence in their work:

[My research mentor is] somebody who I know is pushing me, who’s behind me … who’s willing to put her neck out for me as a student, and she’s been really supportive … [she] helped me realize that I can do this type of work, even though I don’t have much experience in it. Even though I might be the youngest person in the department, it’ll be okay.—URM female

Research mentors were also important sources of inspiration. Students often said they felt proud of working with and being mentored by researchers they looked up to in their fields. They mentioned the importance of receiving affirmation, feedback, and support from people they admire. One student described feeling valued by her research mentors’ encouragement and support, noting that, “The work that comes out of their lab, it’s some of the best in the nation, and they make sure that my work is good.” Another student described a research mentor as someone who took time out of his schedule to check in with her regularly and noted how she values the insights of a mentor who “has done it”:

He’s just been really great in terms of asking me what I want to do, where I want to go with my life and how I can reach those goals. And just being a sounding board for, “Well, I’m thinking about this. Should I do this or that?” That kind of mentorship or someone that’s older and has done it and has seen things change.—non-URM female

Collaboration with what one student called “world-class scientists” not only facilitated high-quality mentoring relationships for students, but also played a critical role in building their confidence and fostering in them a more salient sense of scientific identity. This is described in the following section.

### Influence of Mentoring on Scientific Identity

When students were asked if they identified as scientists, they overwhelmingly said they did. Sixteen of 24 respondents indicated that, at least to some extent, they saw themselves as scientists. However, students’ explanations of their identities indicated that they varied along a spectrum from weaker, less salient or undeveloped, to stronger, more salient and fully developed, scientific identity. Students with weaker scientific identities were those who expressed hesitation or lack of clarity about this identity or said they felt they were not quite scientists, but on their way. These students described themselves as “science-interested,” “scientist in training,” or as “leaning toward” being a scientist. Some said outright they did not identify as scientists at all. Students with stronger scientific identities were quicker and more confident in their expression of this identity and often articulated specific qualifications they felt made them scientists. These students also commonly expressed that being a scientist was an important part of their overall identity.

The salience of students’ scientific identities corresponded to a number of qualities in their mentoring relationships. While most students did not explicitly draw this connection, the ways students with strong or weak scientific identities talked about mentorship and identity were similar, reflecting commonalities in the experiences of students in these groups. Students with more salient scientific identities tended to have more established, longer-term mentoring relationships. They described their mentors as sources of support, and guidance, but also as collaborators who facilitated opportunities for their growth. One URM female student with strong scientific identity salience described receiving strong, long-term mentorship from her research advisors and collaborators. This mentorship was characterized not only by support and encouragement, but by feedback and accountability:

If my work’s not good, they don’t tell you like, “Oh, this is horrible.” They say, “This is how you should make this [better].” And you’re kinda seeing eyebrows like [look of concern], but they tell me how to make it much better.

Receiving direct support and feedback from her mentors gave this student better research skills and more confidence in her abilities as a scientist. She described how taking on more independent work in the lab, with this support, enabled her to call herself a scientist.

In contrast, students with less salient scientific identities described fewer mentoring relationships overall. These relationships tended to be initiated by students as necessary, with little long-term commitment, collaboration, or academic accountability. For example, one female URM student with weaker scientific identity salience mentioned a few mentors during her interview, including a postdoctoral researcher, a CSS staff member, and a professor. Throughout her discussion of these individuals, she described how each of her mentoring relationships were self-initiated and based on provision of advice without accountability. In describing the lack of accountability, the student expressed some frustration, saying, “I have to do it myself.” While this student described a number of successes in her work including “having basically all the postdocs wanting me to do their work,” she was concerned about calling herself a scientist, saying “I don’t think I’m there yet” and “I tend to doubt myself a lot.”

While most students received some form of research mentorship, those who did not also tended to express less salient scientific identities. One non-URM male student described that “mentors are one of the areas that I have a scarcity of resources.” The mentors he discussed during his interview were teaching mentors who he described as providing a “voice of reason” and guidance when they had academic concerns. While this student was engaged in research, he did not consider his research collaborators to be mentors. Instead, he said, he only went to them when he had work-related questions. And while this student was very science-interested and had been accepted into multiple PhD programs in a STEM field, he explained that he felt “limited” in his “ability to really be a scientist.”

Some students described drastic changes in their scientific identity over their time during college and in the CSS program, typically moving from initially having no sense of scientific identity to now fully and confidently self-identifying as a scientist. Others described more subtle progressions over time. A few students said they came to college and the CSS program already feeling like scientists:

I guess I do consider myself a scientist. I think I can think critically, find a problem, have a reasonable hypothesis, test data about it. I think I’ve felt that way for a while. Like, entering the program I felt that way.—Non-URM female

This, however, was not a common experience. Most students entered the program with a weaker sense of scientific identity, but their conceptualization and expression of this identity developed over time. Many students gained confidence through their STEM coursework and ultimately found fulfillment in their scientific identities as they applied their learning through lab work or other research. Indeed, students overwhelmingly described scientific identity as achieved through experiences outside the classroom. In particular, students emphasized the distinction between scientific “thinking,” which many felt was insufficient to identify oneself as a scientist, and scientific “action.” This was true not only for students who strongly identified as scientists, but also for those who expressed a weaker scientific identity:

I don’t currently identify as a scientist. It’s something I want to work towards. Right now, I feel like a student. I have a long path ahead of me … I’ve got the thinking down, and I’ve got the – I guess the – I’ve got the thinking down; I just don’t have the action down yet.—Non-URM male

A female student elaborated on this, saying that it took her a year and half of doing her own research and taking STEM courses to feel strong in that identity:

At this point I’m definitely a scientist. I would say the point is when I started to write up my own results and have a paper and general ideas about my own research project and being able to present at a lab meeting … actually doing research.—Non-URM female

For many students, this emphasis on scientific action—versus some other qualification—helped them reject the notion that scientists were “elite.” One non-URM female student described how “a lot of times, people have this – they think ‘scientist’ is like a high-up word. But I think when you’re doing … any type of science, you’re a scientist.” For others, however, the emphasis on scientific action led to a weakening of formerly held scientific identity. One student described how, despite identifying strongly as a scientist during childhood, he now felt more limited in that identity:

On one hand, yes. It’s one of those, like, forever things. It’s always been a part of the identity … since I was 10 or 11 … On the other hand, not really, you know? Like, I’m doing some science, but I’m not publishing daily. You know, there’s a whole bunch of other things that I’m doing that I think limit my ability to really be a scientist.—Non-URM male

These students felt they were on their way to becoming scientists, but had not taken the necessary steps to achieve that identity:

I don’t think I’m not a scientist, but I think I haven’t quite reached the point where I’m comfortable saying, “Hey, I’m a [scientist] who studies [this],” that sort of step there. I don’t think I’ll reach that point until either I publish my first paper or complete grad school or something.—URM male

While most students emphasized the role of research in fostering their scientific identities, they generally did not draw an explicit connection between research experiences, identity, and mentorship. Instead, students tended to describe their scientific identities as achieved through independent work and reflection. However, our interview data suggest that mentorship did play a key role in strengthening students’ scientific identities through the research process. Mentors—particularly research mentors—facilitated and supervised formative experiences, supported students as they pushed their own limits, and served as examples of what it meant to be a scientist.

Many students saw their mentors as sources of encouragement and support as they pursued more independent work, giving them confidence in their abilities as scientists:

What I’m most proud of is just taking up this really big task [in the lab] and feeling completely confident in doing it. And then also just writing my thesis … I’ve never written a paper to the point that it’s publishable. I’ve written them turning in for school and stuff, but this paper is so good. And I had my advisors working with me the whole time.—URM female

For students with stronger scientific identity, mentors not only encouraged them as they pursued independent work, but facilitated the opportunities to do so. This was especially the case for research mentors. For example, one student discussed the postdoctoral researcher who mentored her in a lab, saying, “She’s just really mentored me from the very beginning … She’s held my hand the entire way.” She went on to discuss the role this mentor played in fostering experiences that shaped her scientific identity, including publication opportunities.

She asked me if it was okay if she put my name on the publication. I don’t know. Just feeling very included, like, “Hey, I’m gonna be published,” is, I think, the most satisfying. Everything that I’ve done leading up to the moment makes me an actual scientist.—URM female

For other students, while research mentors played a less direct role in these formative experiences, they inspired students’ aspirations toward scientific identity. Many students aspired to not only meet the expectations of their research mentors, but to match them in their work. They described feeling proud and satisfied after presenting their research at conferences or when networking with colleagues, a role they often saw their mentors taking on.

I’m the only undergraduate on my study … So, just like satisfying. It’s like I presented on it and I did the entire thing by myself, and I was able to answer all of the questions … So I think that was the biggest achievement that I’ve made in research was just being able to contribute and answer questions just as well as my PI [principal investigator] could.—Non-URM female

For other students, interacting with research mentors challenged their previous notions of what it meant to be a scientist. One non-URM female student discussed how her identity evolved from being a “hard scientist” to what she called a “business scientist”:

I feel like I was a hard scientist coming in, in terms of experience and hypothesis and testing and all that. And now I think I’ve moved more towards – this isn’t even a word – but a business scientist … using technology to solve business needs and testing that and seeing what can we do that’s different … And I don’t think I recognized that that too is science. Even though I’m not a chemist in a lab and pouring chemicals and boom-reaction-done … So I consider myself a scientist in that aspect, but maybe not the traditional research scientist working in a lab.

This student described how, through her research experiences and engagement with mentors, her scientific identity developed in unanticipated ways. While other students retained more traditional definitions of scientists, their understandings of the scientific community, and their own role in it were similarly shaped by their research and mentoring experiences.

## Discussion

This analysis adds to the growing body of research around the influence of mentorship for STEM students and sheds light on potential pathways through which mentorship influences scientific identity. Specifically, we provide additional insights into characteristics of successful mentoring relationships, scientific identity processes for STEM students, and the role of mentors in shaping and verifying scientific identity.

### Characteristics of Successful Mentoring Relationships

Our qualitative analysis was able to shed light on the complexities of mentoring relationships which are not fully understood through quantitative research on mentoring (Robnett et al., 2018). We found that students characterized their mentoring relationships as positive or successful when they offered a balance of personal closeness and academic accountability, when they built on shared values and identities, and when they were multidirectional in nature, with both mentor and mentee sharing responsibility for setting mentorship goals and initiating contact. This aligns with previous work on the theoretical attributes of successful STEM mentoring relationships (Mondisa & McComb, 2015; Pfund et al., 2016). Our finding about the unique role of research mentors in particular reinforces literature on the value of mentors in developing students’ research skills and self-efficacy (Pfund et al., 2016).

We also saw that having a shared identity with a mentor was important to students, but to varied extents. Like Hernandez et al. (2017), we found that while demographic similarity to a mentor played a role in the success of mentoring relationships, the more salient factor was the extent to which protégés saw themselves as similar to their mentors in terms of their broader values and perspectives. While these perspectives may have related to the backgrounds of both mentors and protégés, not all students felt that having a matched-background mentor was critical. However, other students preferred mentors that shared their background. Syed et al. (2012), in their study of preferences for matched-ethnic mentors among URM adolescents in STEM, similarly found individual differences in preferences for matched-background mentors and suggested the need for more tailored mentoring approaches. Additionally, while not the focus of our research, we saw some indication that informal relationships were preferable to formal mentoring relationships. Students who described meeting mentors “organically,” rather than outside the context of CSS or some other program, tended to have longer-term, bidirectional (equally initiated by both mentor and protégé) relationships.

Equally important to understanding successful mentoring relationships is understanding unsuccessful or negative mentoring (National Academies of Sciences, Engineering, and Medicine, 2019). In our study, students did not elaborate on negative mentoring experiences. Instead, students tended to either discuss positive mentorship experiences in-depth or to mention mentors only briefly, with short descriptions of mentor roles but no rich discussion of mentoring history or relationships. One student did not discuss mentorship at all, saying he had no mentors. It could be that students who deemphasized the role of mentoring did so because they had little to say about mentors, or because they had little experience with mentoring. Alternatively, students may have felt that negative mentoring experiences were normal or may have felt it was important to withstand such mentoring in order to gain perceived benefits, such as professional connections, recommendations, or affiliation with a lab. It could also be that they were aware of data collectors’ affiliations with CSS and UNC-CH as an institution and therefore were hesitant to go in-depth on negative mentoring experiences. Regardless, our limited findings about these experiences underscore the importance of qualitative research that is more directly focused on understanding negative mentoring in-depth.

### Scientific Identity Processes for STEM Students

We found that scientific identity was expressed along a spectrum for our students and that it evolved over time. This echoes what has been described in the literature. For example, in their longitudinal qualitative study of out-of-class experiences of graduating STEM majors, Thiry, Laursen, and Hunter (2011) found that students’ scientific identities evolved over time and that the extent to which students expressed any sort of professional identity varied across the sample. In the same way, a study of professional identity formation among engineering undergraduates found that while some students fully considered themselves engineers, others felt their identities as engineers were still in progress (Eliot & Turns, 2011). In our sample, we similarly saw that some students felt very little sense of a professional identity (as a scientist or otherwise), whereas others strongly saw themselves as scientists.

Interpreting our results through the lens of identity theory provides additional insights into the processes by which students developed, refined, and expressed their scientific identities. For students in our sample, identity shaped and was shaped by their actions as scientists. Students with less salient scientific identities described being less involved in research, publication, or independent scientific work. While these activities may not have been of interest to students, it is also possible that they chose to deprioritize engagement in these activities as a way to communicate other, non-scientific elements of their identities. Similarly, students with strong scientific identities, who were engaged in these activities, may have done so as a way to signal their identity to others. At the same time, scientific engagement may have reinforced students’ confidence, skills, and self-efficacy to promote a more salient scientific identity.

### How Mentoring Influences Scientific Identity

Identity theory also provides insights into how mentoring influenced scientific identity processes for students in our sample. We found that mentors were an important part of identity verification for students in our sample (Burke & Stets, 2009; Stets et al., 2017). For some students, there appeared to be little discrepancy between how they saw themselves and perceived others to see them. For example, for students who saw themselves as scientists, having mentors who supported this identity reinforced it and may have made it more salient. However, other students experienced discrepancies between their own and others’ views of their identity. For example, we discussed one student in our sample who received wide approval on her scientific work and described “having all the postdocs wanting me to do their work.” However, this student did not see herself as a scientist. While this was initially a surprising finding, interpreting it through identity theory sheds light on the possible underlying process—by emphasizing her non-scientist identity and deemphasizing her own scientific work, this student may have been trying to counteract discrepancies between her own and others’ view of her scientific identity. By deemphasizing her own scientific identity, she may have been working to reduce the distress of others’ expectations, which contradicted her own.

While students in our sample did not explicitly discuss experiences of being told they were “bad” scientists, there may have been students who identified strongly as scientists, but encountered others who disagreed. According to identity theory, these students would, in an effort to reassert their identities, more strongly exhibit “scientific” behavior which might include research, publication, presentation, or even talking about science. Understanding this process is particularly important for research with URM students, who may seek to overemphasize their scientific identities in response to identity threat or stereotype threat. However, others have found that stereotype threat may actually reduce scientific identity, and that the perceived authenticity or sincerity of mentoring relationships may change the influence of identity verification (Fries-Britt & White-Lewis, 2020; Smith et al., 2015). Given the socially embedded nature of identity processes, future research should seek to better understand how discrimination and stereotypes may influence identity formation, verification, and salience for URM and female STEM students.

Scholars have emphasized the need for understanding how mentor-mentee similarity may influence outcomes for STEM students (National Academies of Sciences, Engineering, and Medicine, 2019). Our findings suggest that shared identities may indeed shape the relationship between mentorship and scientific identity. For students in our sample, mentors served as an example for navigating career challenges and aspirations within a given combination of intersectional personal identities (e.g., gender, racial, and ethnic identities). Students expressed that being able to relate to mentors helped them feel close to them; this may have made both students and mentors more willing to engage in the relationship. Shared identity may facilitate social community, which has been highlighted as an element of successful mentoring programs by Mondisa and McComb (2015). According to their findings, mentoring programs establish social community through repeated “dynamic, multidirectional interactions among like-minded individuals” (Mondisa & McComb, 2015 p.159), which can create successful, long-term relationships characterized by social support and learning. Indeed, we saw that students in our sample preferred relationships that involved active communication, partnership, growth, and two-way communication, all building on a sense of shared identity and relatability. This may have supported their scientific identity formation. However, students expressed that striking a balance between intimacy, shared identity, and accountability with their mentors was challenging.

Social community may have also been established through peer or near-peer mentoring, including mentorship from older or more experienced CSS and STEM students. A few students in our sample mentioned receiving informal peer mentorship, and some noted that they served as formal peer mentors for the CSS program and for STEM courses at UNC-CH. These discussions were uncommon, so we were unable to fully consider how these processes may have shaped social community. However, others have found that peer and near-peer mentoring programs can facilitate coping, support, and positive academic outcomes for STEM students (Garcia-Melgar & Meyers, 2020; Zaniewski & Reinholz, 2016). It is also possible that non-mentoring peer or near-peer interactions fostered by CSS participation may have facilitated social community and enhanced the success of mentoring.

Finally, mentor identity was just one of several characteristics that influenced how mentorship fostered scientific identity for students in our sample. We found that students valued mentors who collaborated with them on research and that these relationships may have fostered a stronger sense of scientific identity for protégés. Indeed, the literature suggests that research experiences play a critical role in shaping students’ scientific identities (Merolla & Serpe, 2013). A qualitative study with graduating STEM majors found that among students who described identifying as a scientist, most attributed that feeling to out-of-class experiences, including research (Thiry et al., 2011). Further, a longitudinal study of undergraduates across the USA found that participants’ research involvement predicted their scientific identities 2 years later and that the association between research and scientific identity was mediated by science self-efficacy (Robnett et al., 2015). A 2011 review of professional identity development in higher education similarly found that research and practice played a key role in development of professional identity for students and noted that “collaborative, dialogic learning from practice” was a consistently enabling factor (Trede et al., 2012). Our findings similarly suggest that it is not only exposure to research experiences, but also the nature of such experiences in combination with collaborative mentorship, that plays a critical role in how such experiences facilitate identity formation.

### Limitations

There were limitations to our research. First, although there was variation in students’ levels of research experience, the CSS program has a research requirement and emphasizes STEM research broadly in its activities. This may have led students to discuss research mentoring relationships more extensively than others. Second, limiting our sample to students in their senior year of college means we were unable to explore mentoring and scientific identity among students at other places in their academic trajectories. This also means our sample was limited to students who successfully completed the CSS program and excludes students who may have dropped out of the program at an earlier stage, who may have different mentoring experiences. However, limiting our sample to students in their senior years of college enabled us to include students who were able to provide richer insights given their longer duration of college and mentoring experiences. This also enabled us to answer questions about the role of mentoring as students prepare to transition out of college. Our findings are also context-specific, which may limit their generalizability to other populations of STEM students. However, the goal of qualitative research such as ours is to understand in-depth, situated experiences of individuals, rather than to generate widely generalizable findings.

Third, the data used for this analysis were quite structured, which limited our ability to deeply understand mentoring relationships in this sample. In particular, interviews provided limited information on how students identified mentors and how (and by whom, in some cases) mentoring relationships were initiated. This limited our ability to fully assess differences between formal and informal mentoring relationships. Further, our study did not involve the collection of data from mentors themselves, which may have provided valuable insights into the context and characteristics of mentoring relationships. However, others in the field have demonstrated that undergraduate students’ perceptions of their mentoring relationships quantitatively predict scientific identity, whereas mentors’ perceptions of those same relationships do not (Robnett et al., 2018).Finally, the balance of URM and non-URM students in our sample allowed us to begin assessing how these students compare in their experiences with mentoring and scientific identity. However, because we only had interviews from 12 senior URM students and our data were fairly structured, we were not able to achieve saturation when assessing the unique experiences of URM students. Because the overall goal of our study was to evaluate a program, saturation of themes was not the primary goal or expectation when recruiting participants. Despite this, our analysis provides rich insights into the experiences of members of two underrepresented and high-priority groups in STEM—URM students and female students—with mentoring and scientific identity.

## Conclusions

This analysis highlights the importance of research mentorship in fostering scientific identity and increases our understanding of how different types of mentoring experiences may contribute to development of future leaders in STEM. Specifically, we contribute to the mentoring literature with our findings that mentees desire not just support, but accountability from mentors; that mentorship outside of research contexts may only be partially successful in shaping scientific identity; and that, for URM and female students in particular, shared mentor-mentee identity and values play a key role in relationship success. We also draw on identity theory to explain how research engagement shapes scientific identity salience for STEM students, while simultaneously allowing students to communicate their scientific identities to others. Mentors serve as key players in identity verification processes and may serve to reinforce students’ own identities or prompt them to counteract discrepancies.

In elaborating on existing mentoring theory and literature, we have also identified a number of considerations for STEM enrichment programs, particularly those focused on fostering scientific identity for URM and other students. First, our results reveal a need for more tailored mentoring approaches. While we found that shared values and identity were important for scientific identity formation, as Syed et al. (2012) emphasize, not all URM students want a matched-background mentor, and programs should not assume they do. Where possible, mentoring programs should consider student preferences when formally matching students to mentors.

Second, given the role of research in expressing and reinforcing scientific identity, there is a need to integrate mentorship with high-quality, collaborative research experiences. For students in our sample, research collaborators often naturally became mentors for students, but this was not always the case. Programs may want to consider ways to more intentionally link research and mentorship to ensure research experiences offer students opportunities for collaboration, dialog, and professional identity formation in addition to the research skills gained.

Finally, STEM enrichment programs should consider how they can better equip students to form mentoring relationships on their own. While formal mentoring programs have demonstrated benefits, some students in our study indicated they preferred mentoring relationships that were less formal or that they wanted more mentoring, but not all had the skills to initiate these relationships. Programs should incorporate strategies to help students navigate how to ask for and organize mentorship based on their own preferences.

These findings also raise a number of additional questions which should be considered by researchers in the field. First, while peer mentorship was not the focus of this analysis, our findings suggested it may play a role in scientific identity formation. However, it is unclear the extent to which peer mentorship may operate differently or through different mechanisms than non-peer mentorship; this warrants further, more focused exploration. Second, this is one of a number of cross-sectional studies exploring these relationships. Additional longitudinal research would be useful in assessing causal relationships between mentorship and scientific identity or other outcomes, and could allow for assessment of longer-term outcomes.

## Data Availability

Interested parties should contact the lead author for access to the data and materials.

## Abbreviations

CSS: Chancellor’s Science Scholars
STEM: Science, technology, engineering, and mathematics
UNC-CH: University of North Carolina at Chapel Hill
URM: Underrepresented minority
